# Supplementary material for: New Anti-Glycative Lignans from the Defatted Seeds of Sesamum indicum
Source: Molecules. 2023 Feb 28;28(5):2255. doi: 10.3390/molecules28052255 (PMC10005424; doi:10.3390/molecules28052255)
Supplement: Supplementary file 1 [file molecules-28-02255-s001.zip › molecules-2210858-supplementary.pdf]

Supplementary Material

New Anti-Glycative Lignans from the Defatted Seeds of *Sesamum indicum*

Gyeong Han Jeong † and Tae Hoon Kim *

Department of Food Science and Biotechnology, Daegu University, Gyeongsan 38453, Republic of Korea; jkh4598@kaeri.re.kr
* Correspondence: skyey7@daegu.ac.kr; Tel.: +82-53-850-6533
† Current address: Research Division for Biotechnology, Advanced Radiation Technology Institute (ARTI), Korea Atomic Energy Research Institute (KAERI), Jeongeup 56212, Republic of Korea.

Contents

Figure S1. ^{1}H NMR spectrum of compound **1** in CD_3OD.

Figure S2. ^{13}C NMR spectrum of compound **1** in CD_3OD.

Figure S3. ^{1}H-^{1}H COSY spectrum of compound **1** in CD_3OD.

Figure S4. HSQC spectrum of compound **1** in CD_3OD.

Figure S5. HMBC spectrum of compound **1** in CD_3OD.

Figure S6. Expanded key HMBC correlation of compound **1** (H-7' to C-5).

Figure S7. NOESY spectrum of compound **1** in CD_3OD.

Figure S8. HRFABMS spectrum of compound **1**.

Figure S9. CD spectrum of compound **1**.

Figure S10. ^{1}H NMR spectrum of compound **2** in CD_3OD.

Figure S11. ^{13}C NMR spectrum of compound **2** in CD_3OD.

Figure S12. ^{1}H-^{1}H COSY spectrum of compound **2** in CD_3OD.

Figure S13. HSQC spectrum of compound **2** in CD_3OD.

Figure S14. HMBC spectrum of compound **2** in CD_3OD.

Figure S15. NOESY spectrum of compound **2** in CD_3OD.

Figure S16. HRFABMS spectrum of compound **2**.

Figure S17. CD spectrum of compound **2**.

Figure S18. ^{1}H NMR spectrum of compound **3** in CD_3OD.

Figure S19. ^{13}C NMR spectrum of compound **3** in CD_3OD.

Figure S20. ^{1}H NMR spectrum of compound **4** in CD_3OD.

Figure S21. ^{13}C NMR spectrum of compound **4** in CD_3OD.

Figure S22. ^{1}H NMR spectrum of compound **5** in CD_3OD.

Figure S23. ^{1}H NMR spectrum of compound **6** in CD_3OD.

Figure S24. ^{13}C NMR spectrum of compound **6** in CD_3OD.

Figure S25. ^{1}H NMR spectrum of compound **7** in CD_3OD.

Figure S26. ^{13}C NMR spectrum of compound **7** in CD_3OD.

Figure S27. ^{1}H NMR spectrum of compound **8** in CD_3OD.

Figure S28. ^{13}C NMR spectrum of compound **8** in CD_3OD.

Figure S29. ^{1}H NMR spectrum of compound **9** in CD_3OD.

Figure S30. Chemical structures of the new compounds **1** and **2** isolated from defatted *Sesame* cake.

Table S1. AGEs formation inhibitory effects on the fraction from defatted *Sesame* cake.

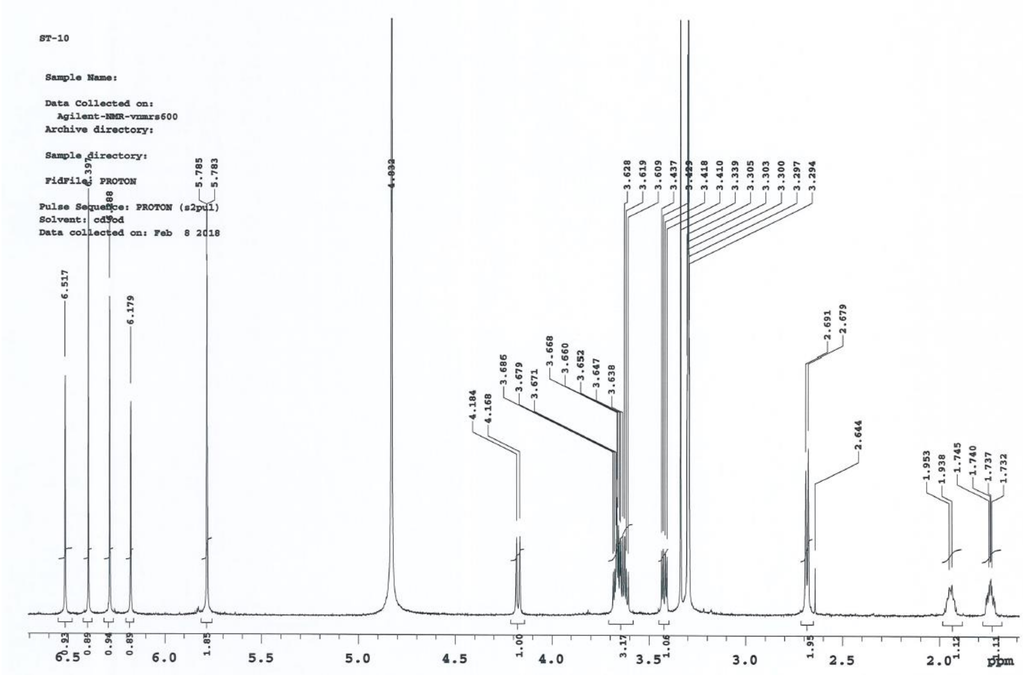

Figure S1. ^{1}H NMR spectrum of compound **1** in CD_3OD.

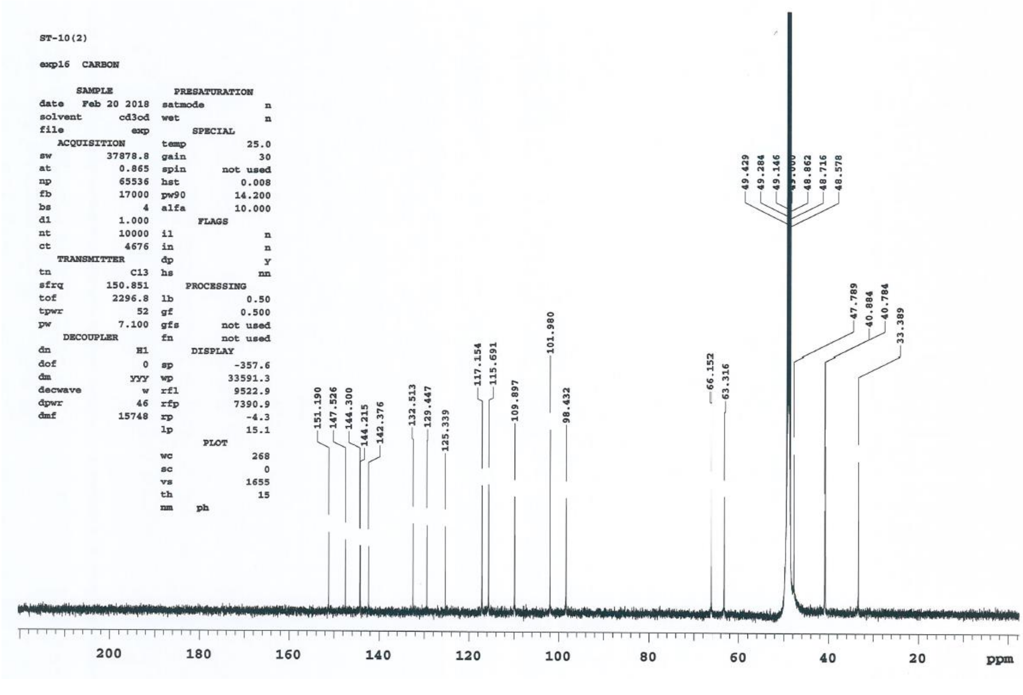

Figure S2. ^{13}C NMR spectrum of compound **1** in CD_3OD.

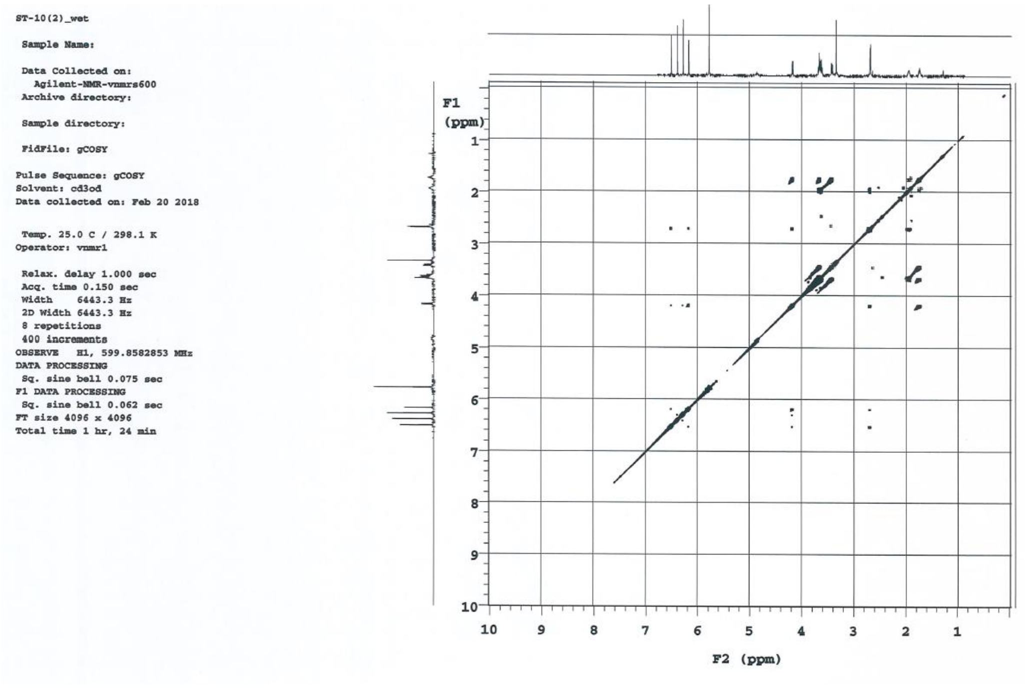

Figure S3. ^{1}H-^{1}H COSY spectrum of compound **1** in CD_3OD.

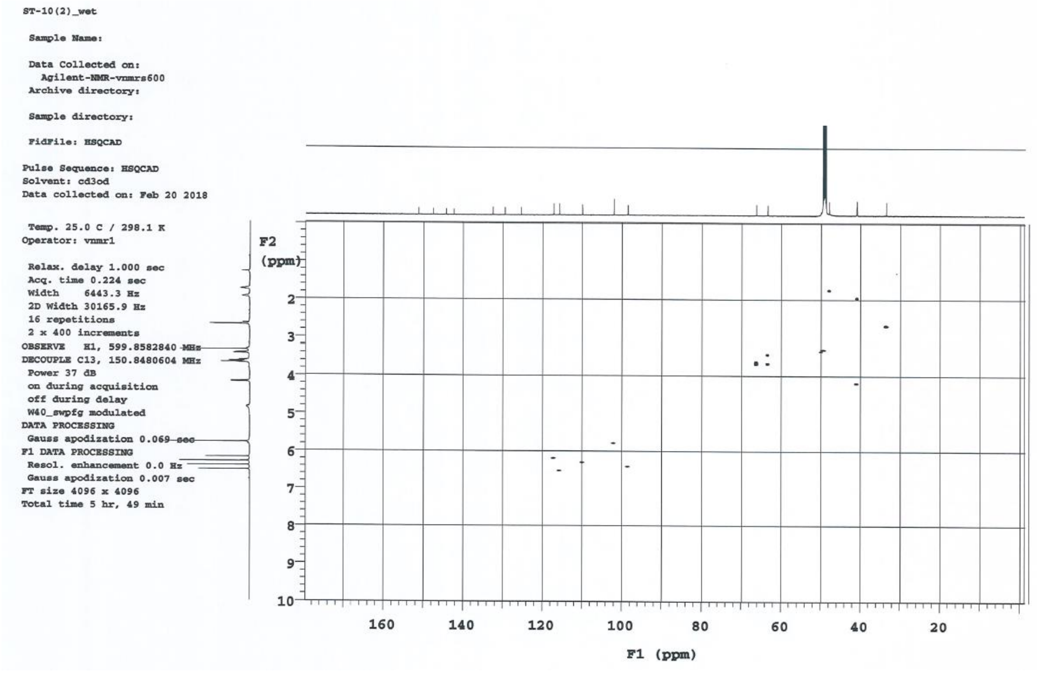

Figure S4. HSQC spectrum of compound **1** in CD_3OD.

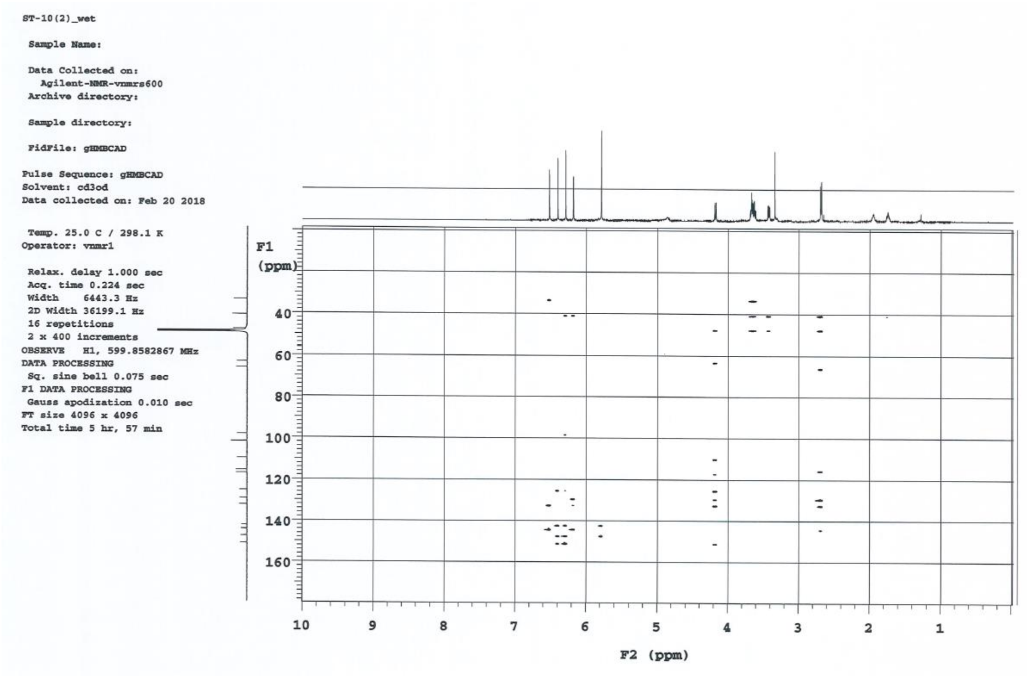

Figure S5. HMBC spectrum of compound **1** in CD_3OD.

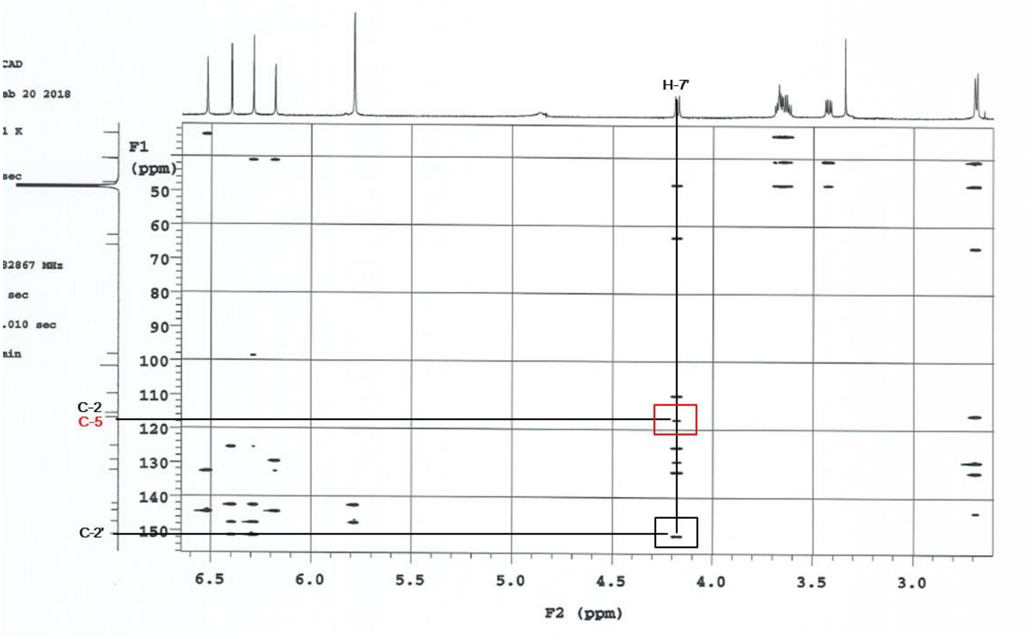

Figure S6. Expanded key HMBC correlation of compound **1** (H-7' to C-5).

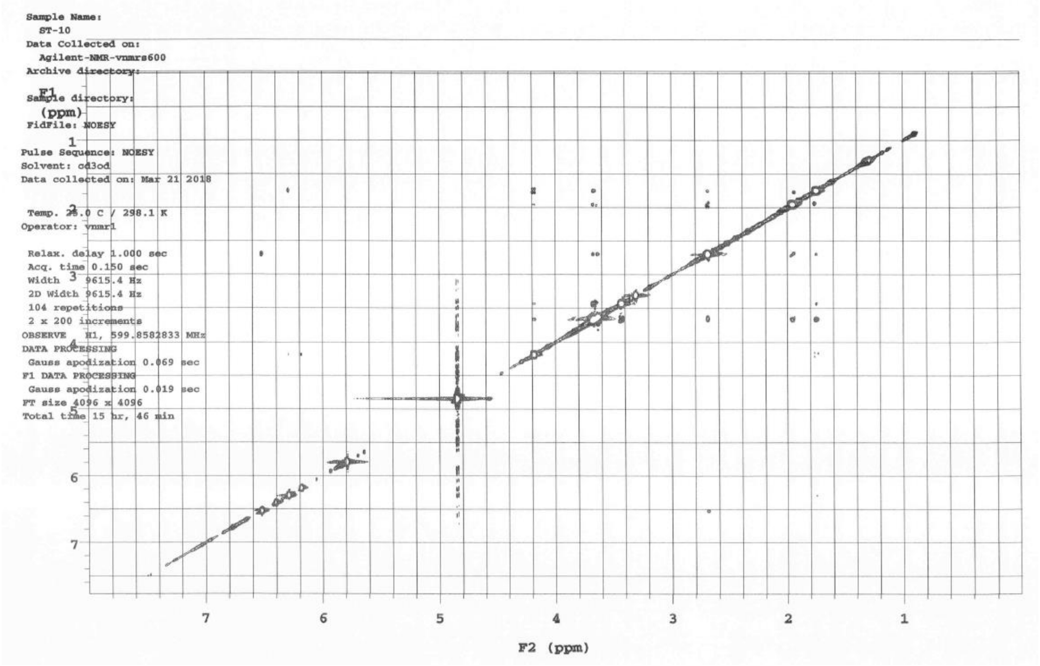

Figure S7. NOESY spectrum of compound **1** in CD_3OD.

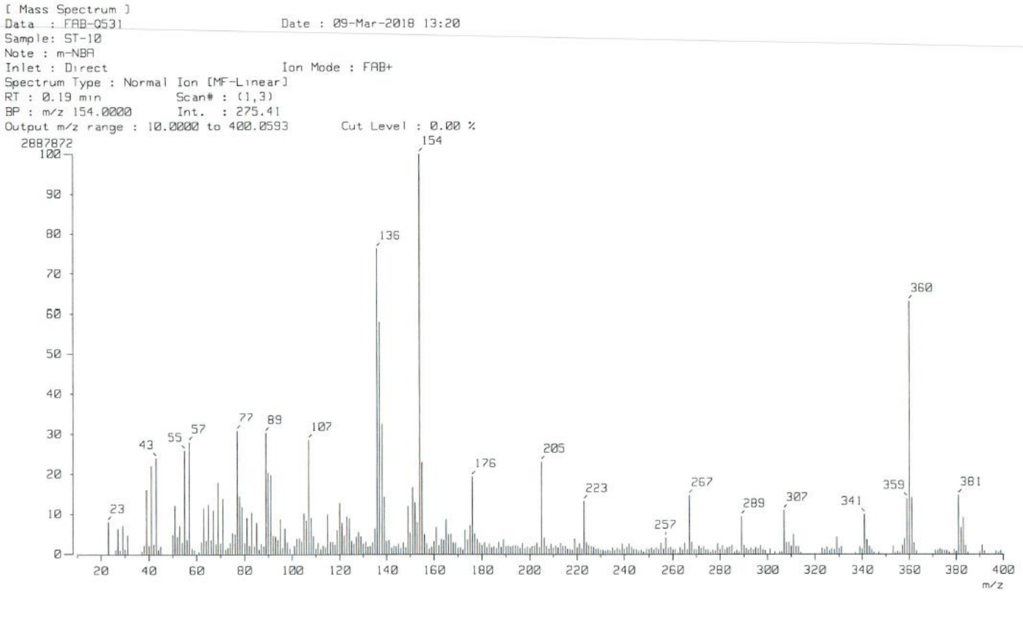

[Elemental Composition]
Page: 1

Data : FAB-Q542
Date : 09-Mar-2018 18:14
Sample: ST-10
Note : m-NBA
Inlet : Direct
Ion Mode : FAB+
RT : 0.47 min
Scan#: (18,27)
Elements : C 100/0, H 100/0, O 10/0
Mass Tolerance : 20ppm, 5mmu if m/z < 250, 10mmu if m/z > 500
Unsaturation (U.S.) : -0.5 - 30.0

Observed m/z	Int%	Err[ppm / mmu]	U.S.	Composition
360.1206	100.0	+15.5 / +5.6	19.0	C 26 H 16 O 2
		-0.8 / -0.3	10.0	C 19 H 20 O 7

```
[ Theoretical Ion Distribution ]                                      Page: 1
Molecular Formula : C19 H20 O7
                      (m/z   360.1209, MW   360.3636, U.S. 10.0)
Base Peak :   360.1209, Averaged MW :   360.3613(a),   360.3621(w)

    m/z        INT.
  360.1209 100.0000 **********************************************************
  361.1243  21.3991 ************
  362.1267   3.5754 **
  363.1293   0.4388
  364.1318   0.0451
  365.1342   0.0040
  366.1367   0.0003
```

Figure S8. HRFABMS spectrum of compound **1**.

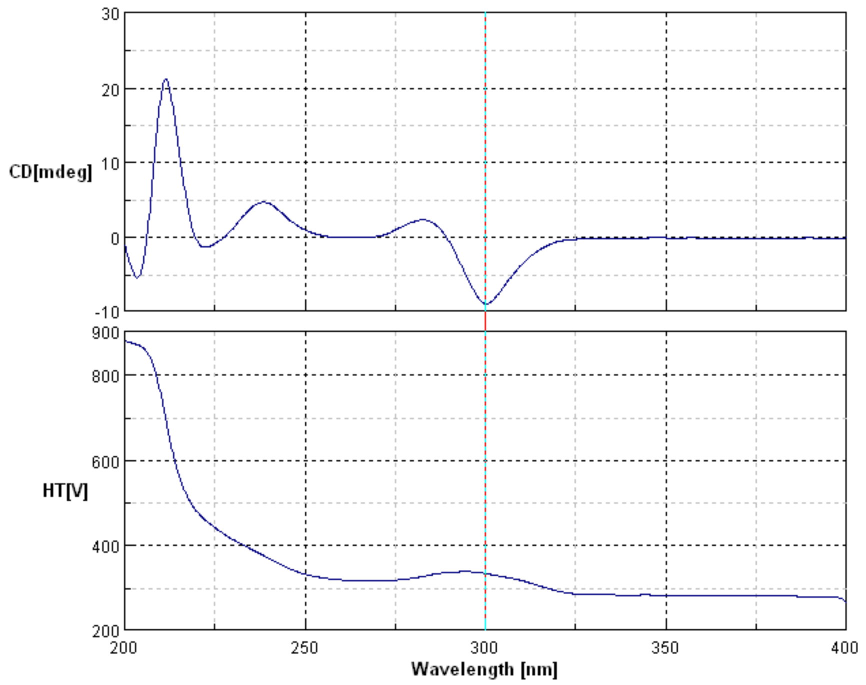

Figure S9. CD spectrum of compound **1**.

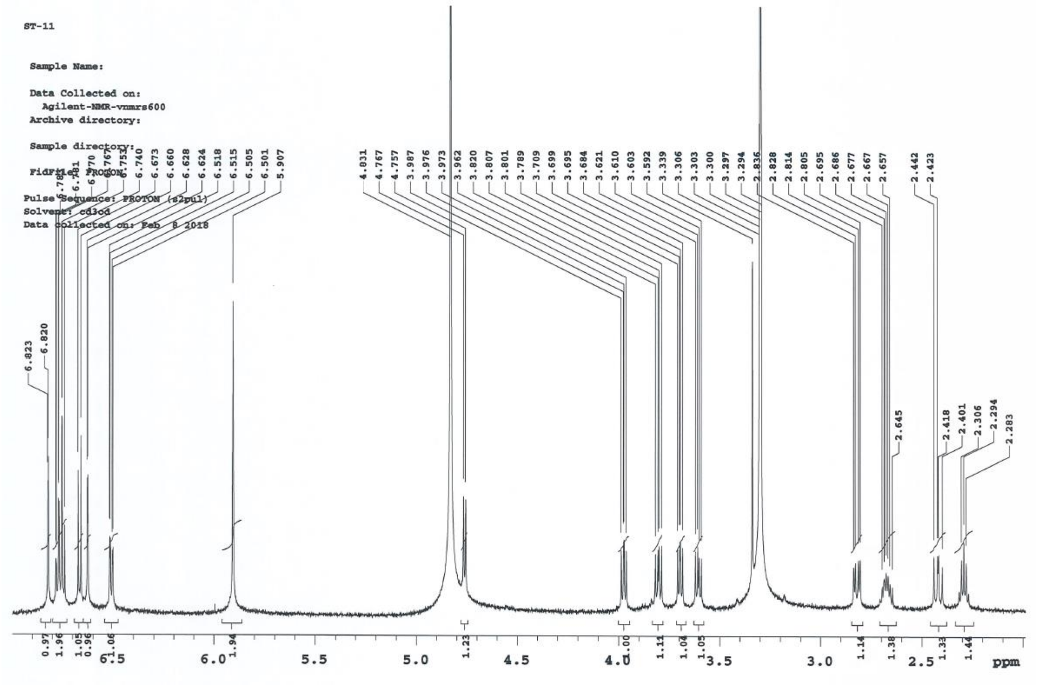

Figure S10. ^{1}H NMR spectrum of compound **2** in CD_3OD.

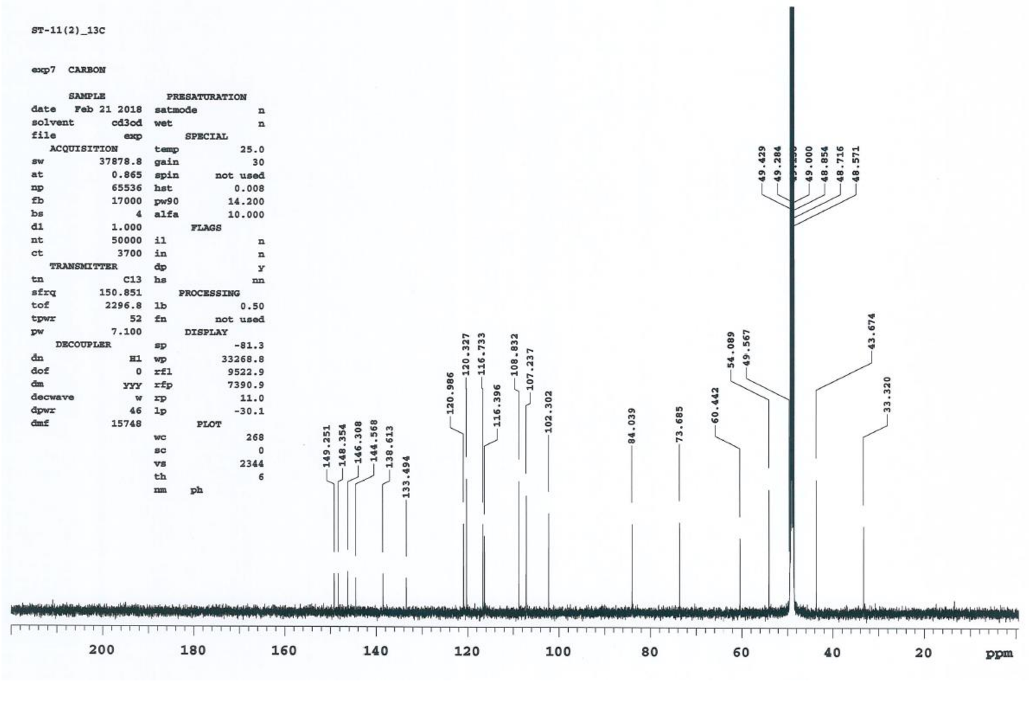

Figure S11. ^{13}C NMR spectrum of compound **2** in CD_3OD.

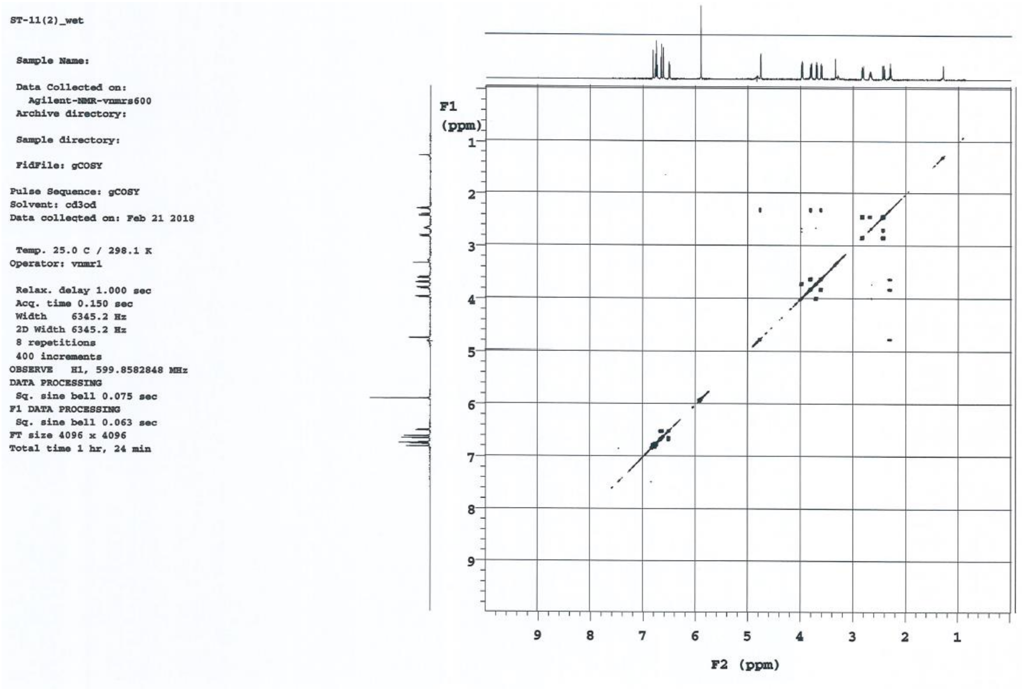

Figure S12. ^{1}H-^{1}H COSY spectrum of compound **2** in CD_3OD.

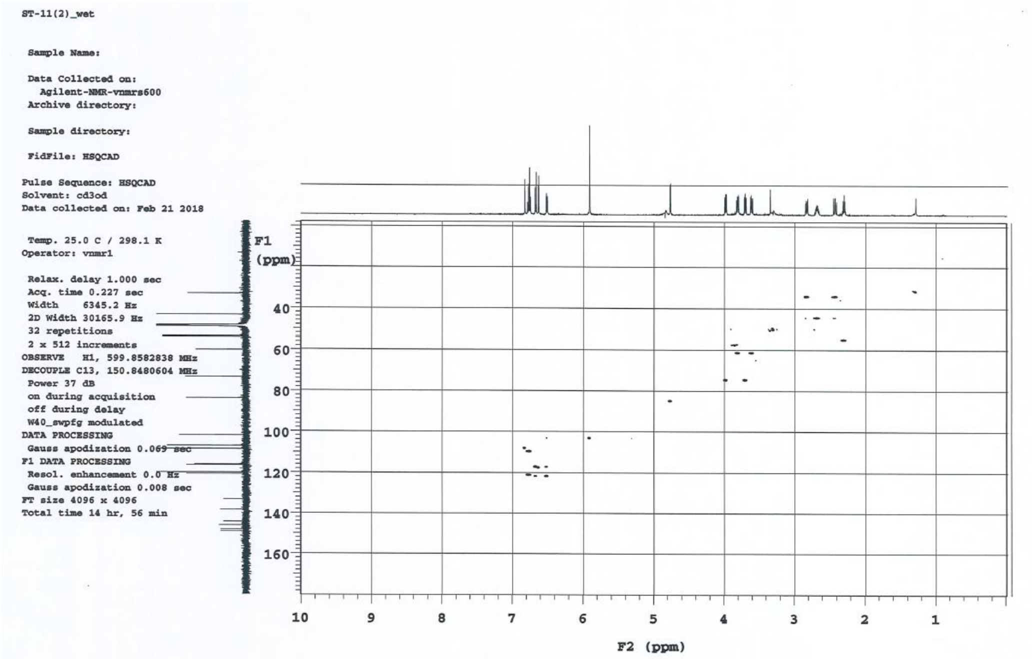

Figure S13. HSQC spectrum of compound **2** in CD_3OD.

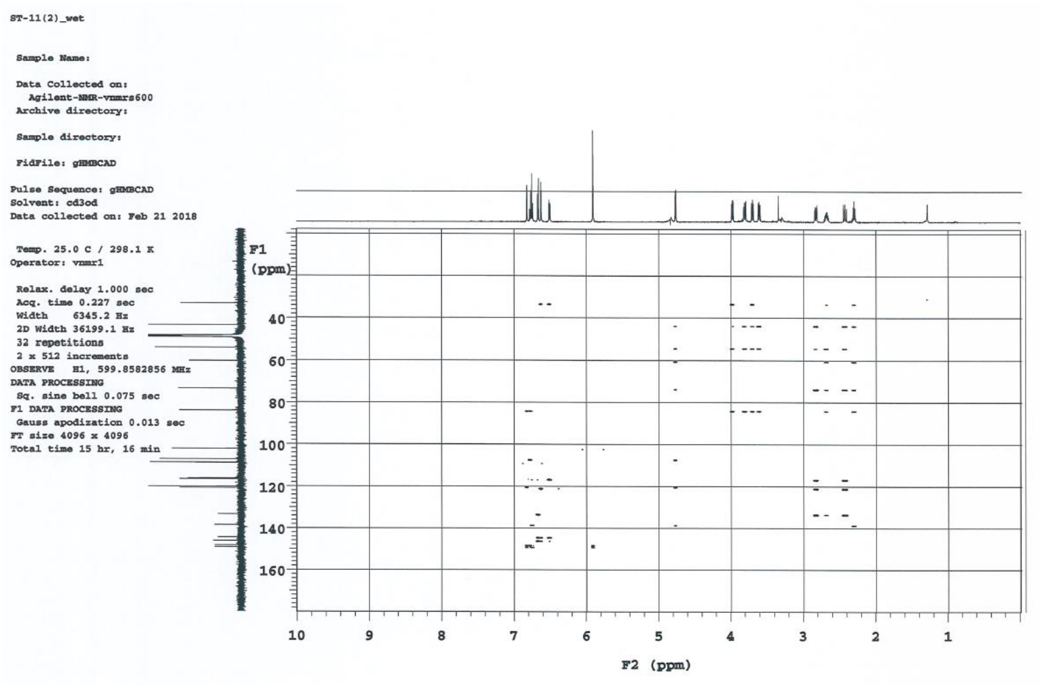

Figure S14. HMBC spectrum of compound **2** in CD_3OD.

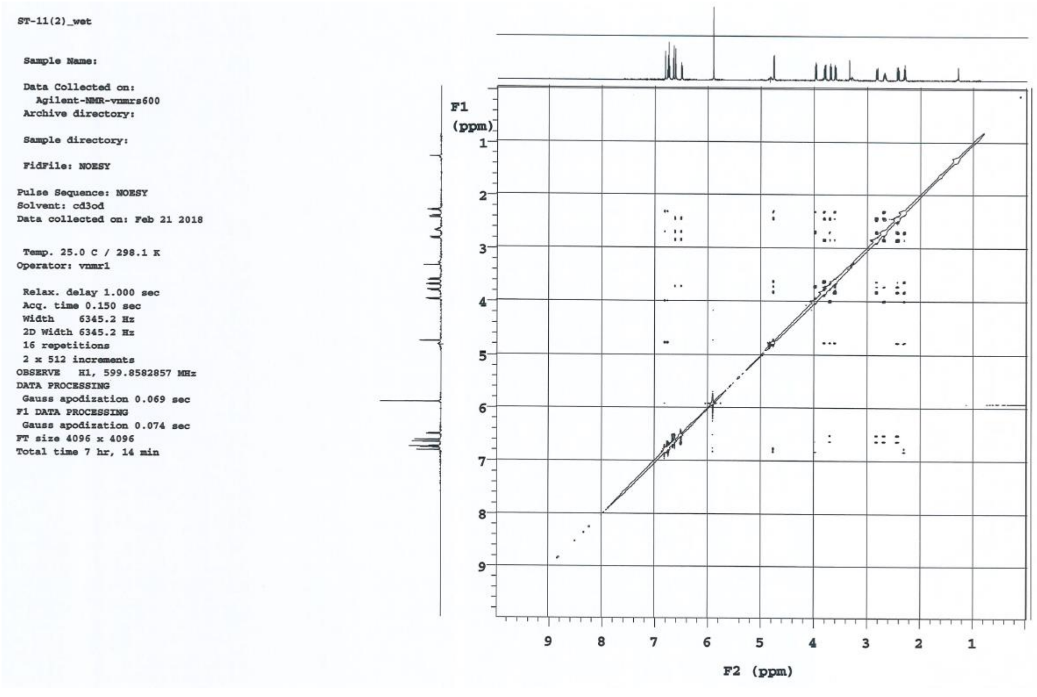

Figure S15. NOESY spectrum of compound **2** in CD_3OD.

[Mass Spectrum]
Data : FAB-Q530 Date : 09-Mar-2018 13:16
Sample: ST-11
Note : m-NBA
Inlet : Direct Ion Mode : FAB+
Spectrum Type : Normal Ion [MF-Linear]
RT : 0.74 min Scan# : (5,6)
BP : m/z 154.0000 Int. : 149.61
Output m/z range : 10.0000 to 380.6737 Cut Level : 0.00 %

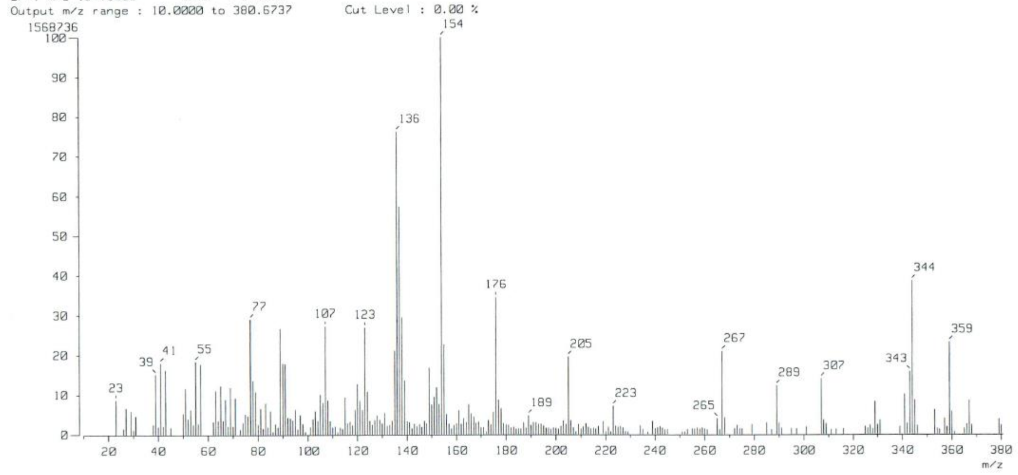


```
[ Elemental Composition ]                                    Page: 1
Data   : FAB-Q544                 Date : 09-Mar-2018 18:21
Sample: ST-11
Note  : m-NBA
Inlet : Direct                    Ion Mode : FAB+
RT : 0.14 min                     Scan#: (2,13)
Elements : C 100/0, H 100/0, O 10/0
Mass Tolerance      : 20ppm, 5mmu if m/z < 250, 10mmu if m/z > 500
Unsaturation (U.S.) : -0.5 - 30.0

Observed m/z  Int%   Err[ppm / mmu]    U.S.  Composition
  344.1262    23.7    +17.7 /  +6.1    19.0  C 26 H 16 O
                       +0.7 /  +0.2    10.0  C 19 H 20 O 6
```

```
[ Theoretical Ion Distribution ]                             Page: 1
Molecular Formula : C19 H20 O6
                    (m/z   344.1260, MW   344.3642, U.S. 10.0)
Base Peak :   344.1260, Averaged MW :   344.3620(a),   344.3628(w)

    m/z       INT.
  344.1260 100.0000 ************************************************************
  345.1294  21.3610 ************
  346.1318   3.3668 **
  347.1345   0.3947
  348.1370   0.0382
  349.1395   0.0031
  350.1420   0.0002
```

Figure S16. HRFABMS spectrum of compound **2**.

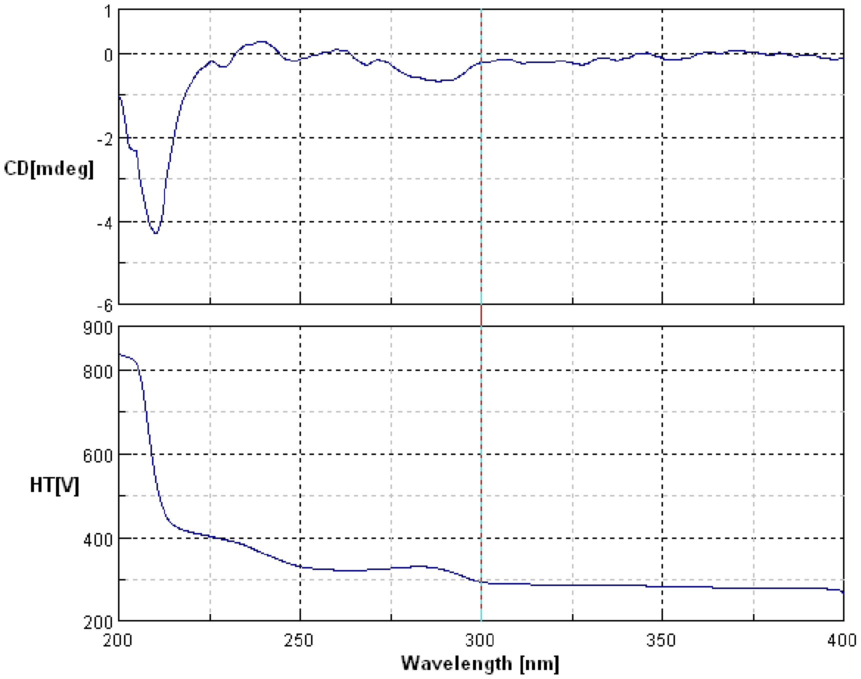

Figure S17. CD spectrum of compound **2**.

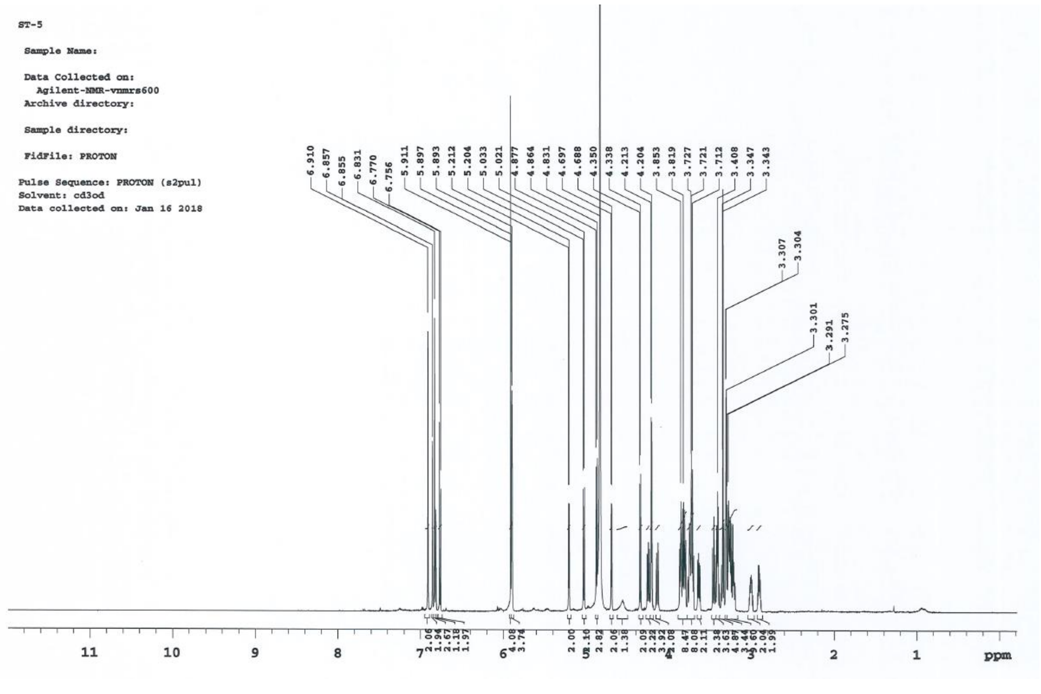

Figure S18. ^{1}H NMR spectrum of compound **3** in CD_3OD.

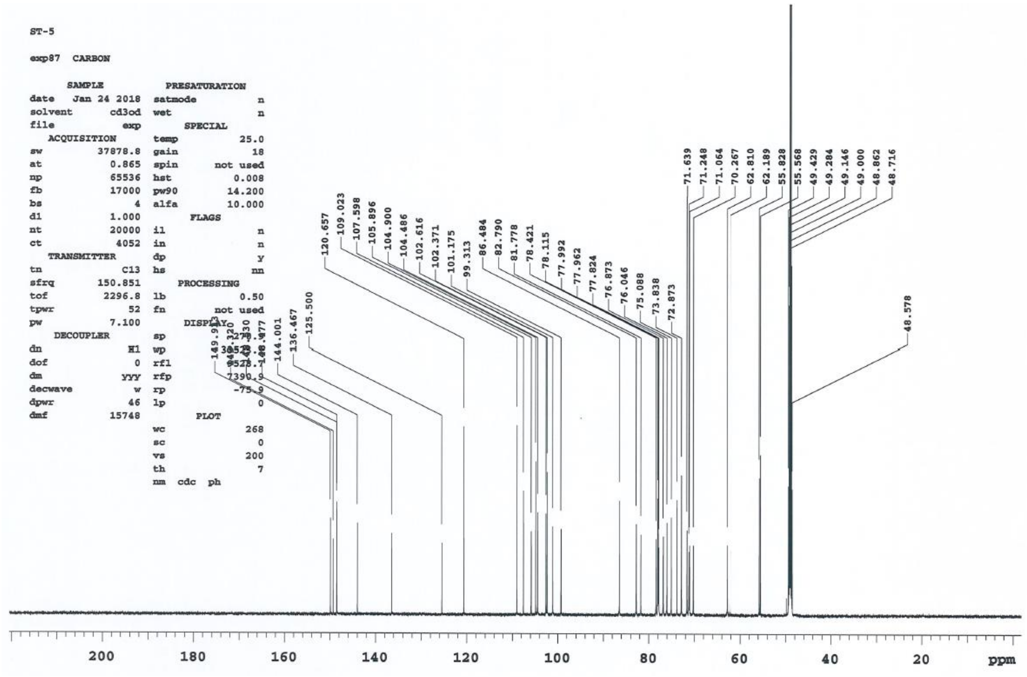

Figure S19. ^{13}C NMR spectrum of compound **3** in CD_3OD.

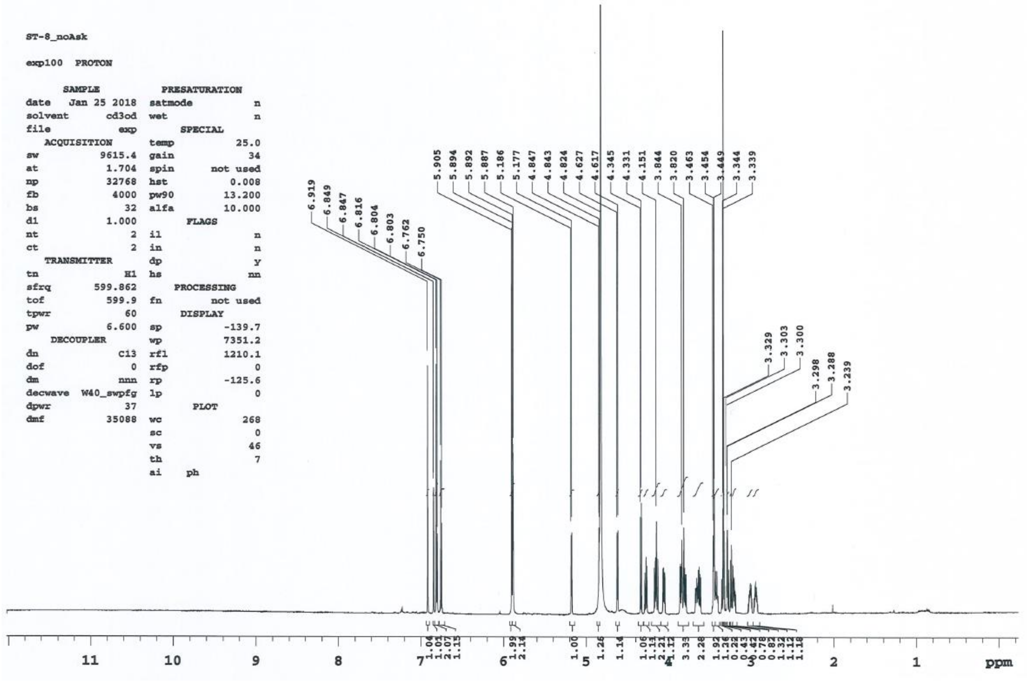

Figure S20. ^{1}H NMR spectrum of compound **4** in CD_3OD.

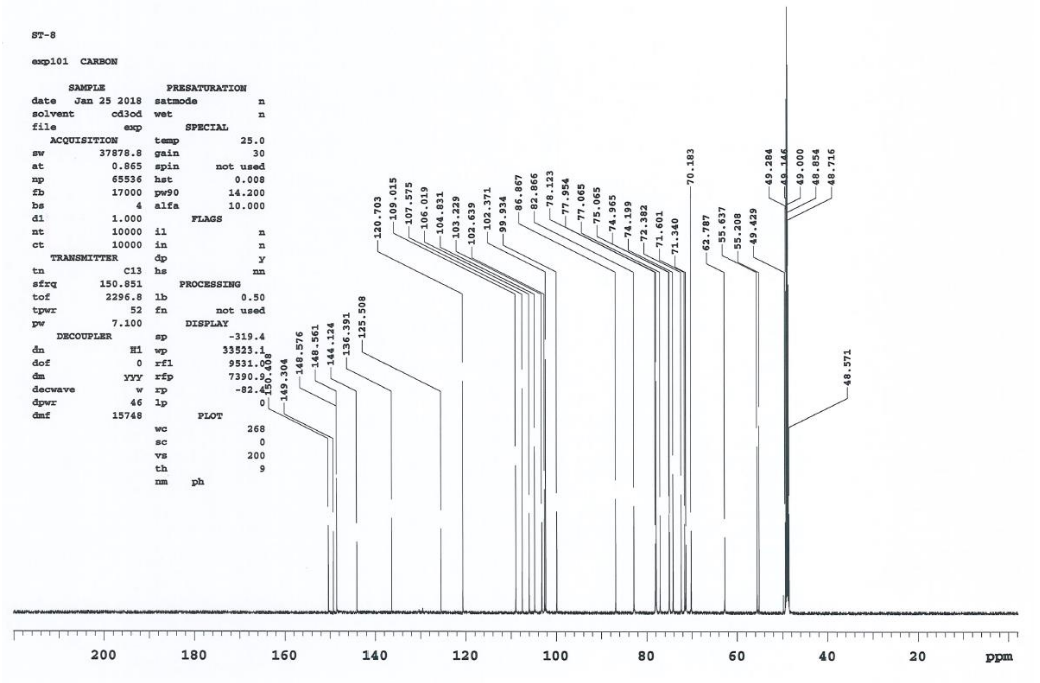

Figure S21. ^{13}C NMR spectrum of compound **4** in CD_3OD.

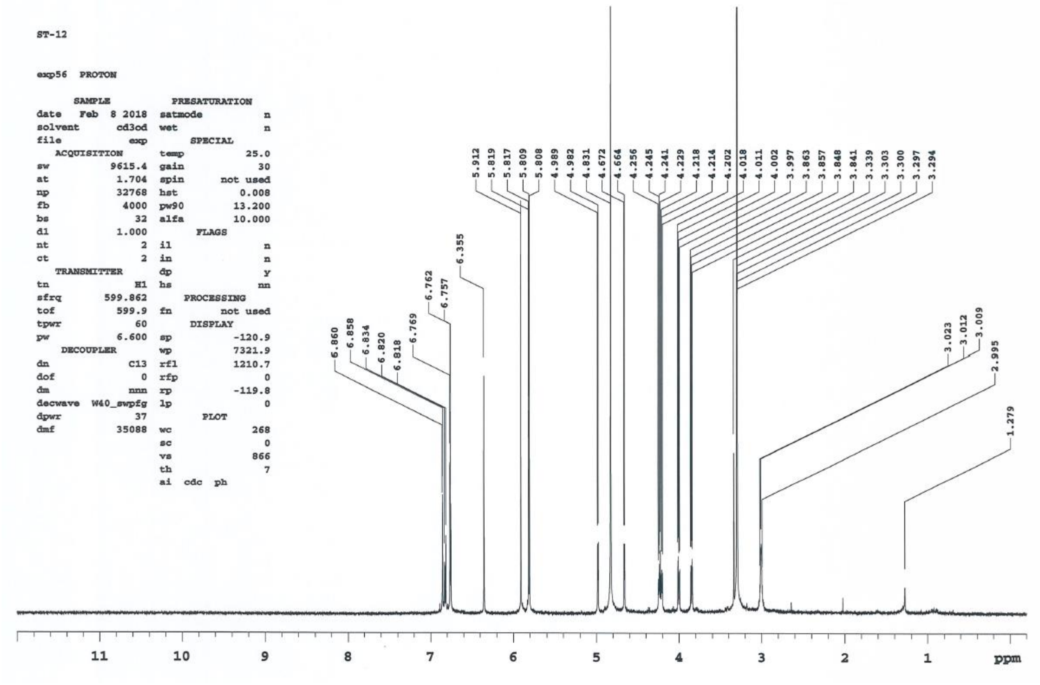

Figure S22. ^{1}H NMR spectrum of compound **5** in CD_3OD.

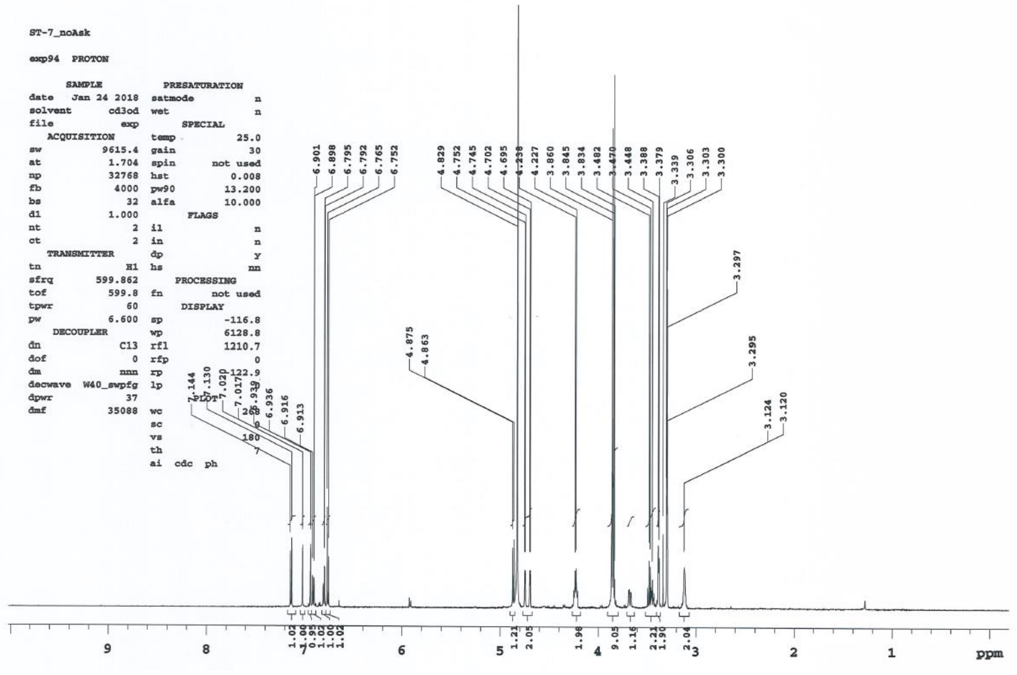

Figure S23. ^{1}H NMR spectrum of compound **6** in CD_3OD.

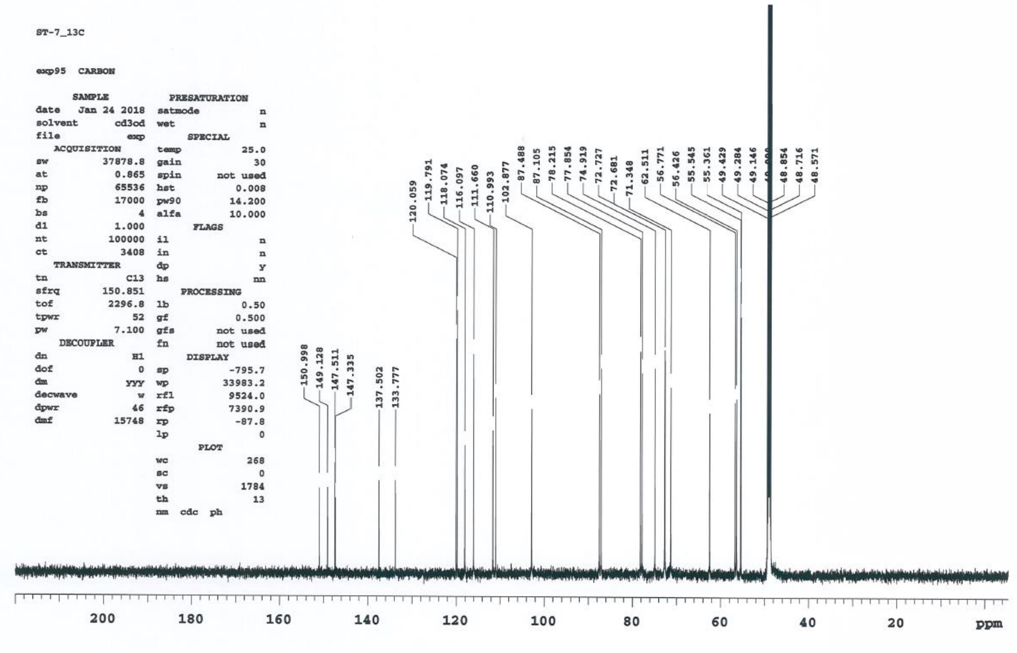

Figure S24. ^{13}C NMR spectrum of compound **6** in CD_3OD.

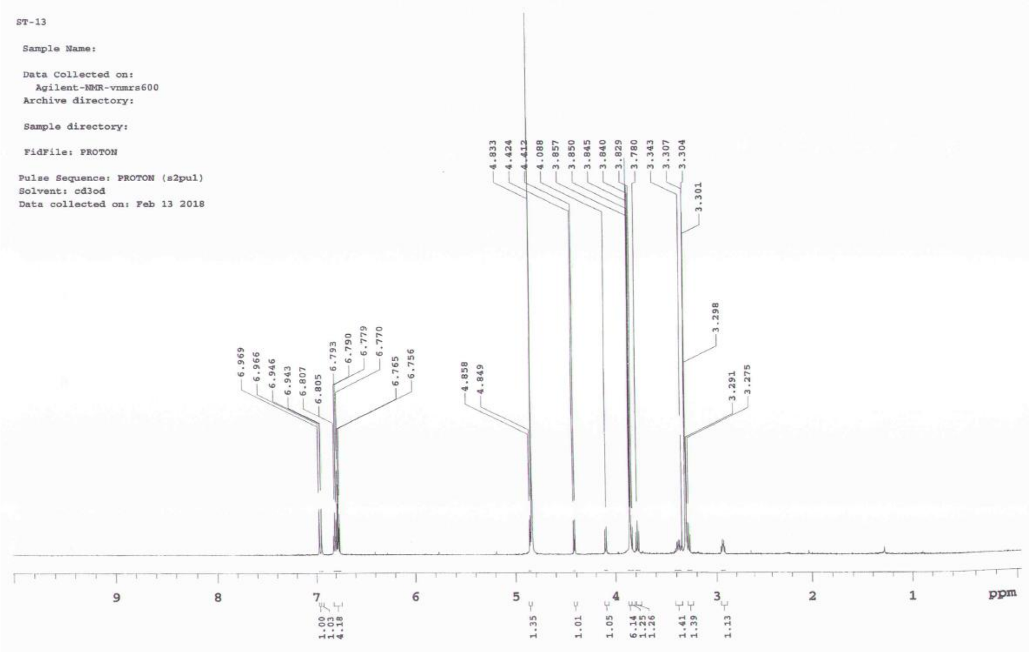

Figure S25. ^{1}H NMR spectrum of compound **7** in CD_3OD.

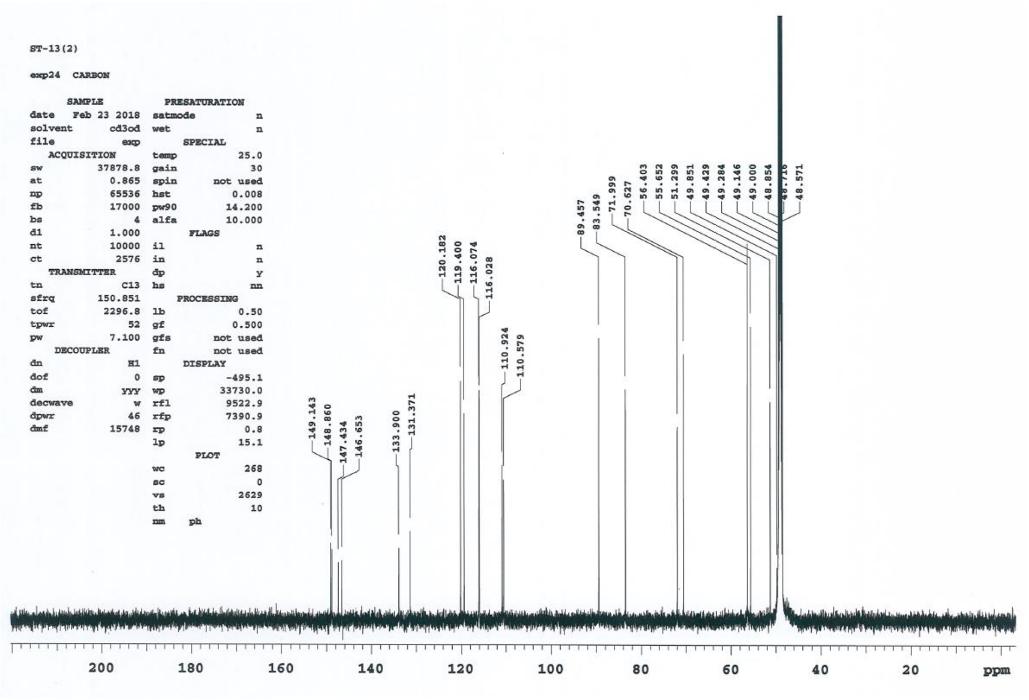

Figure S26. ^{13}C NMR spectrum of compound **7** in CD_3OD.

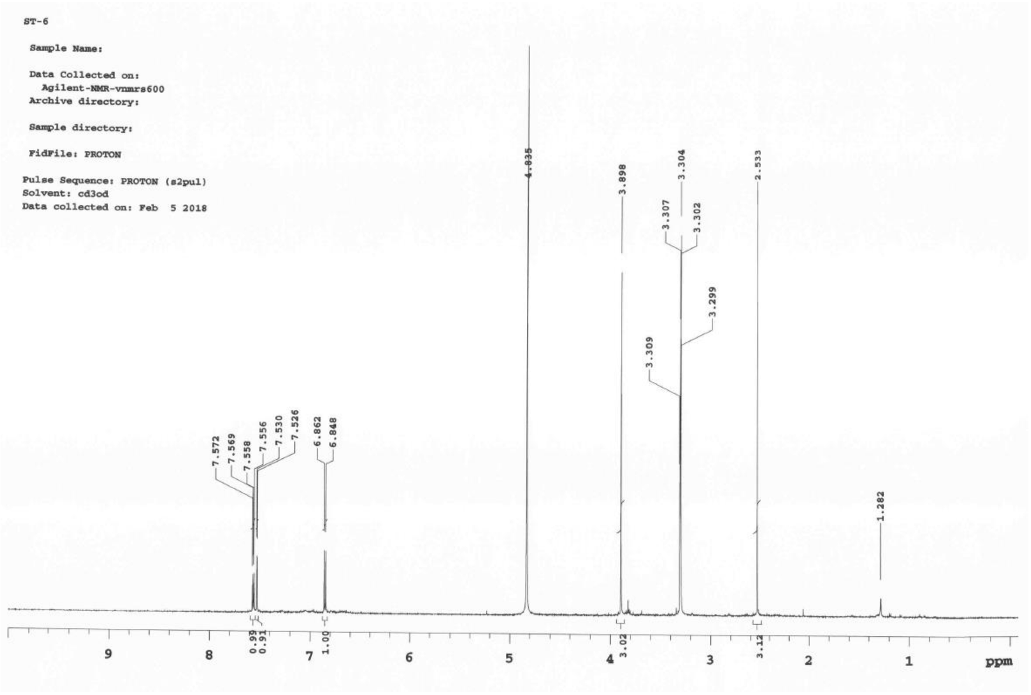

Figure S27. ^{1}H NMR spectrum of compound **8** in CD_3OD.

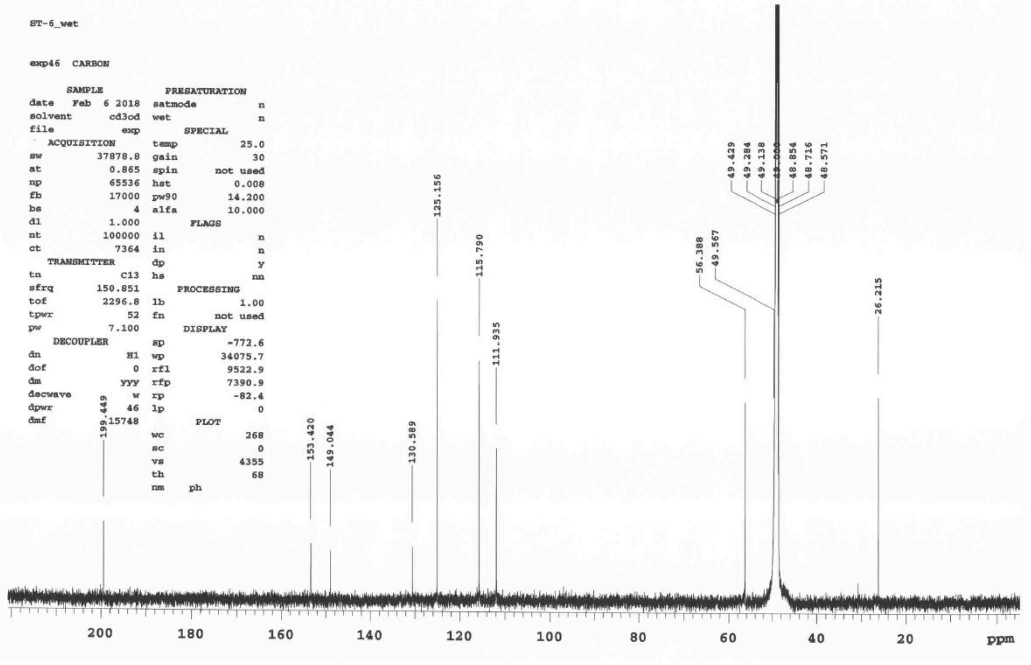

Figure S28. ^{13}C NMR spectrum of compound **8** in CD_3OD.

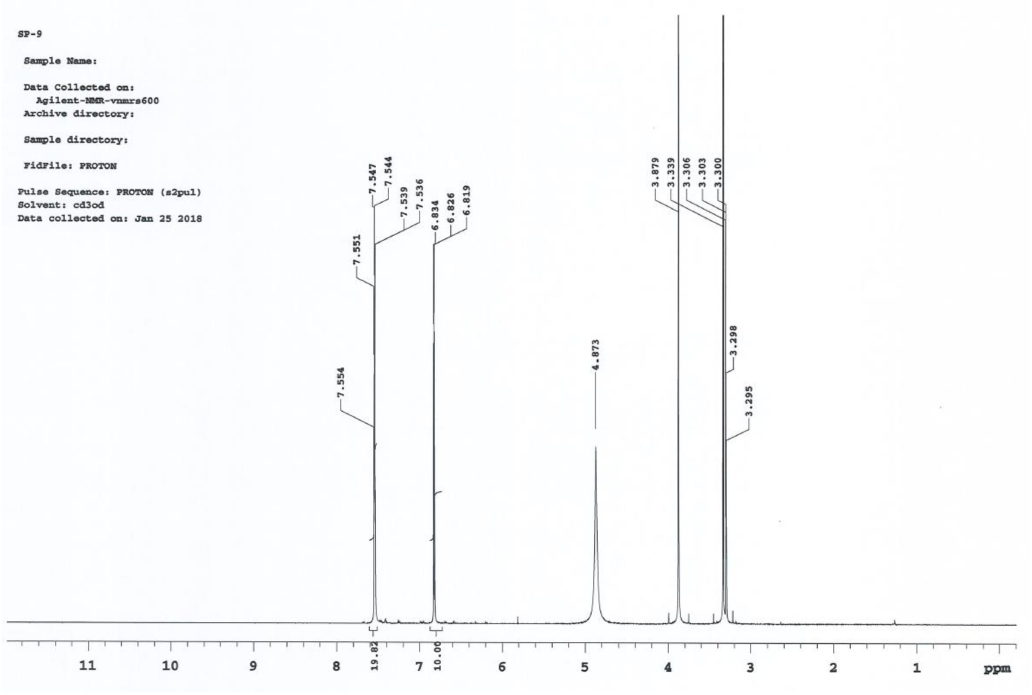

Figure S29. ^{1}H NMR spectrum of compound **9** in CD_3OD.

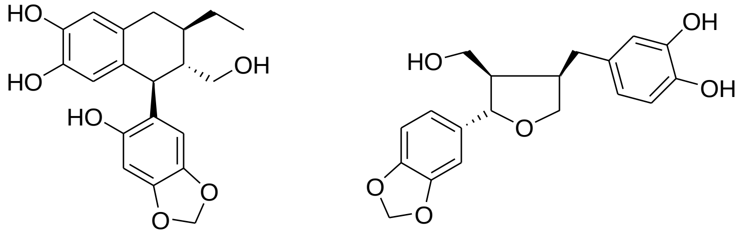

1

2

Figure S30. Chemical structures of the new compounds **1** and **2** isolated from defatted *Sesame* cake.

Table S1. AGEs formation inhibitory effects on the fraction from defatted *Sesame* cake.

Extract and fraction	IC_{50} value (μg/mL)[a]
Hot water extract	236.1±3.1
n-Hexane fraction	>500
EtOAc fraction	154.8±2.4
n-BuOH fraction	368.9±6.8
H_2O fraction	>500
Aminoguanidine[b]	119.7±2.2

[a] All compounds were examined in triplicate experiments. [b] Used as a positive control.
